# Porcine Deltacoronaviruses: Origin, Evolution, Cross-Species Transmission and Zoonotic Potential

**DOI:** 10.3390/pathogens11010079

**Published:** 2022-01-09

**Authors:** Fanzhi Kong, Qiuhong Wang, Scott P. Kenney, Kwonil Jung, Anastasia N. Vlasova, Linda J. Saif

**Affiliations:** 1 College of Animal Science and Veterinary Medicine, Heilongjiang Bayi Agricultural University, No. 5 Xinfeng Road, Sartu District, Daqing 163319, China; fanzhikong110@hotmail.com; 2 Center for Food Animal Health, Department of Animal Sciences, College of Food, Agricultural and Environmental Sciences, The Ohio State University, Wooster, OH 44691, USA; Kenney.157@osu.edu (S.P.K.); jung.221@osu.edu (K.J.); vlasova.1@osu.edu (A.N.V.); saif.2@osu.edu (L.J.S.); 3 Department of Veterinary Preventive Medicine, College of Veterinary Medicine, The Ohio State University, Columbus, OH 43210, USA

**Keywords:** porcine deltacoronaviruses, origin, evolution, cross-species transmission, zoonosis

## Abstract

Porcine deltacoronavirus (PDCoV) is an emerging enteropathogenic coronavirus of swine that causes acute diarrhoea, vomiting, dehydration and mortality in seronegative neonatal piglets. PDCoV was first reported in Hong Kong in 2012 and its etiological features were first characterized in the United States in 2014. Currently, PDCoV is a concern due to its broad host range, including humans. Chickens, turkey poults, and gnotobiotic calves can be experimentally infected by PDCoV. Therefore, as discussed in this review, a comprehensive understanding of the origin, evolution, cross-species transmission and zoonotic potential of epidemic PDCoV strains is urgently needed.

## 1. Introduction

Coronaviruses (CoVs) are single-stranded, positive-sense RNA viruses belonging to the subfamily *Orthocoronavirinae* within the family of *Coronaviridae.* They are classified into four genera: *Alphacoronavirus* (α-CoV), *Betacoronavirus* (β-CoV), *Gammacoronavirus* (γ-CoV), and *Deltacoronavirus* (δ-CoV). Coronaviruses are important pathogens of both humans and animals, causing diverse diseases. α-CoVs and β-CoVs infect mammals, while γ-CoVs and δ-CoVs primarily infect birds with some mammalian spill over [1,2,3]. Deltacoronaviruses were detected from the rectal swabs of mammalian species, including Asian leopard cats (*Prionailurus bengalensis*) and Chinese ferret badgers (*Melogale moschata*) in wet markets, during virological surveillance in southern China between 2005 and 2006 [4]. Since then, δ-CoVs have been detected from a wide range of birds and domestic pigs [5,6,7,8]. The *Deltacoronavirus* genus was defined by genomic sequence analysis of both avian and mammalian isolates [7]. The prototype porcine deltacoronavirus (PDCoV) HKU15 strain was reported in Hong Kong in 2012 [7]. However, its pathogenic potential was not recognized until 2014 when it was found to be the cause of pig diarrheic outbreaks, initially on several farms in Ohio, then spreading throughout the United States (US) and globally [9,10]. PDCoV infects the intestinal epithelial cells and causes acute watery diarrhoea, vomiting and dehydration in gnotobiotic and conventional piglets [9,11,12]. PDCoV causes clinical signs indistinguishable from those caused by other porcine enteric CoVs, such as porcine epidemic diarrhoea virus (PEDV) and transmissible gastroenteritis virus (TGEV) [13]. The clinical impact, prevalence, and disease severity of PDCoV in the field are lower than those of PEDV [14]. However, PDCoV has a broader host range and, unlike PEDV and TGEV, it could/can infect multiple species, including pigs, chickens, turkeys, cattle and humans [7,15,16,17,18]. This review focuses on the current knowledge on the origin, evolution, cross-species transmission and zoonotic potential of PDCoV. Additionally, we discuss the potential mechanisms for PDCoV interspecies transmission from birds to pigs, then to humans to help understand common mechanisms for the emergence of novel human CoVs, such as the highly pathogenic severe acute respiratory syndrome CoV (SARS-CoV), Middle East respiratory syndrome CoV (MERS-CoV), and severe acute respiratory syndrome coronavirus 2 (SARS-CoV-2).

## 2. Origin of PDCoV

**Figure 1:**
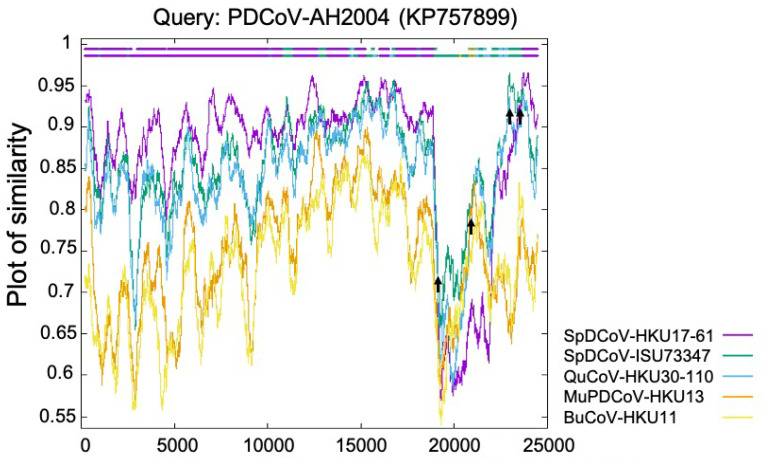
Identification of PDCoV-AH2004 strain as potential recombinant strain using Recombination Identification Program (http://www.hiv.lanl.gov/content/sequence/RIP/RIP.html, accessed on: 28 November 2021). At each position of the window, the query sequence PDCoV-AH2004 was compared with background sequences for 5 avian δ-CoVs (sparrow δ-CoVs HKU17 and ISU73347, quail δ-CoV HKU30, munia δ-CoV HKU13, and bulbul δ-CoV HKU11). The x-axis represents the length of the PDCoV genome, and the y-axis represents the similarity value. When the query sequence is like the background sequence, the homologous regions are indicated as thick dashed lines (of the corresponding color) on the top of the plot. Arrows represent potential recombination breakpoints.

The *Deltacoronavirus* genus was first established by The International Committee on Taxonomy of Viruses (ICTV) in 2012 after the identification of three novel avian coronaviruses (bulbul CoV HKU11, thrush CoV HKU12, and munia CoV HKU13) [6] and seven other novel δ-CoVs (porcine CoV HKU15, white-eye CoV HKU16, sparrow CoV HKU17, magpie robin CoV HKU18, night heron CoV HKU19, wigeon CoV HKU20, and common moorhen CoV HKU21) of birds or pigs [7]. Interestingly, the replicase polyprotein gene (partial cds) of PDCoV HKU15 is closely related (nucleotide identity ≥ 98.88%) to those δ-CoVs (GenBank accession no. EF584909–EF584912) detected in 2006 from the Chinese ferret badgers at wet markets. Similarly, the polyprotein (orf1b, partial cds), spike (S) protein, envelop (E) protein, membrane (M) protein, non-structure protein 6 (NS6), nucleocapsid (N) protein and non-structure protein 7 (NS7) genes of HKU15 share 98.70% nucleotide identity with those of the δ-CoV (GenBank accession no. EF584908) detected from an Asian leopard cat [7]. More importantly, a recent retrospective study has confirmed the presence of PDCoV (CHN/AH/2004) in diarrheic pigs as early as 2004 in Anhui Province, China [19]. These facts suggest that these mammalian δ-CoVs directly or indirectly evolved from avian δ-CoVs. The transmission direction between the small wild carnivores and pigs is still unknown: (1) The wild small carnivore mammals could have been infected through catching and eating δ-CoV-positive birds [20] and could/can act as the intermediate hosts to transmit PDCoV to pigs; or (2) the wild small carnivore mammals could have been infected as they were fed PDCoV-positive pig viscera, which was used as carnivore food in wet markets (Haitao Xiu, personal communication). Natural PDCoV infection of these two mammalian species has not been reported since 2007, even with additional surveillance attempts [7], further supporting the second possibility, although it needs to be validated with more data in the future. Phylogenetic and recombination analyses suggest that PDCoV might have resulted from several recombination events involving δ-CoVs from several avian species, such as sparrow δ-CoVs HKU17 and ISU73347, and quail δ-CoV HKU30 (Figure 1) [8,21]. Recent studies indicate that aquatic birds may serve as natural reservoirs for δ-CoVs, while terrestrial birds and mammalian species may represent spill over hosts [22,23]. Sparrows, one of the most common birds at pig farms during the wintertime, late fall, and early spring, could promote the transmission of δ-CoVs to pigs. For example, bird droppings in the farms or in contaminated feed grains may be consumed by pigs [24]. 

Because the S gene of CoVs is the main genetic determinant of CoV host, tissue, or cellular tropism [25,26,27], Niu et al. [28] and Alhamo et al. [29] tested whether the spike protein or receptor-binding domain (RBD) from the sparrow δ-CoVs could alter PDCoV host and tissue tropism. First, Niu et al. [28] generated an infectious cDNA clone of PDCoV OH-FD22 strain (icPDCoV) and constructed two chimeric icPDCoVs harbouring the spike protein of HKU17 (icPDCoV-S_HKU17_) or the RBD of ISU73347 (icPDCoV-RBD_ISU_). Niu et al. [28] and Alhamo et al. [29] evaluated their replication in the porcine kidney cell line LLC-PK1 and chicken fibroblast cell line DF-1 and performed pathogenesis studies in 4-day-old gnotobiotic pigs, 8-day-old turkey poults, and 11-day-old embryonated chicken eggs (ECEs). Compared with icPDCoV, the two chimeric viruses replicated to lower titers in LLC-PK1 cells. They did not cause clinical signs in pigs and replicated weakly in the nasal cavity but not in the intestines of pigs, whereas icPDCoV replicated mainly in the pig intestines and weakly in the respiratory tract. The two chimeric viruses and icPDCoV did not replicate in DF-1 cells at a multiplicity of infection (MOI) of 0.01 and did not replicate in the turkey poults or ECEs. Thus, these data indicate that the spike protein of sparrow δ-CoV HKU17 and the RBD of sparrow δ-CoV ISU73347 reduced PDCoV replication in pigs, suggesting limited potential for direct cross-species spillover from current sparrow strains to pigs. Recent molecular clock analyses suggest that PDCoVs shared a common ancestor with sparrow CoVs around 1810 and PDCoVs emerged around the 1990s [21]. 

**Figure 2:**
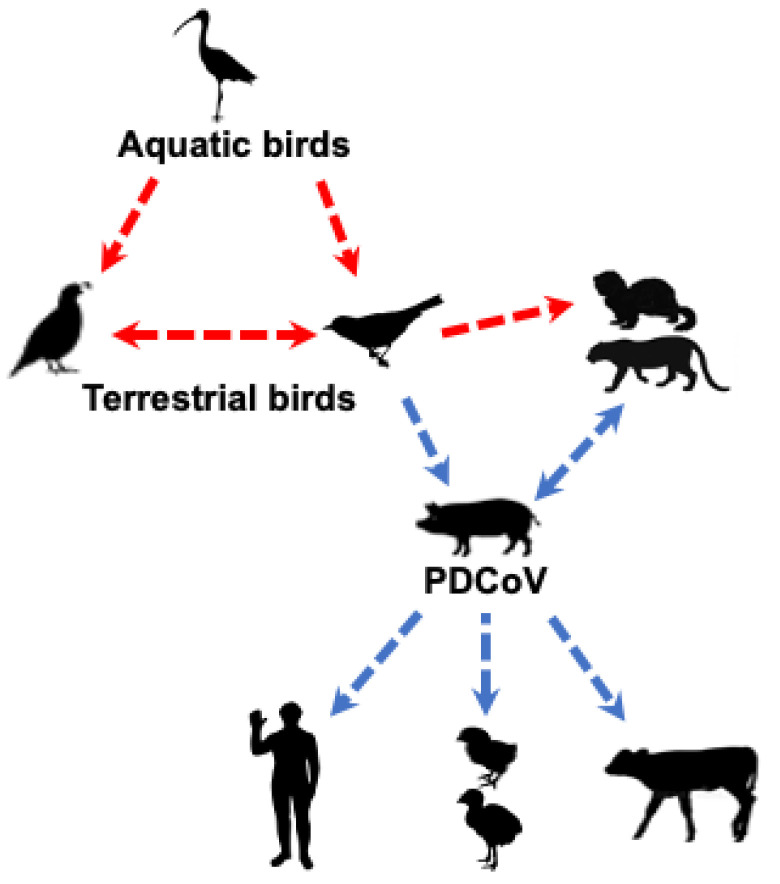
Likely origin and routes of cross-species transmission of PDCoV. The red dashed line indicates potential, but unknown, transmission of δ-CoVs from avian to mammalian species; the blue dashed line indicates potential transmission of PDCoV based on epidemiology or experimental studies.

Collectively, these studies suggest that PDCoV could have originated from terrestrial birds (e.g., sparrows) or less likely from other unknown wild mammalian δ-CoVs (Figure 2). Clearly, a better understanding of PDCoV origin requires detailed epidemiological studies of δ-CoVs in different species of birds and mammals.

## 3. Molecular Epidemiology and Genetic Diversity of PDCoVs

### 3.1. Prevalence of PDCoV in Different Countries

To date, PDCoV has been detected in Hong Kong, the United States, Canada, South Korea, mainland China, Thailand, Vietnam, Laos, Taiwan, Japan, Mexico and Haiti. The first documented PDCoV HKU15-44 and HKU15-155 strains were detected from the faecal samples collected from healthy pigs in 2009–2010 from a large-scale animal surveillance study in Hong Kong [7]. Subsequently, in early 2014, PDCoV associated diarrhoea outbreaks were first reported in three states of the US (Ohio, Iowa and Illinois). Later studies showed that PDCoV had spread to many Asian countries (China, Japan, Korea, Laos, Thailand, and Vietnam) and North American countries (the US, Canada, and Mexico) [10,30,31]. In the US, a retrospective surveillance study using reverse transcription-PCR (RT-qPCR) showed that PDCoV RNA was detected as early as August 2013 in pig faecal samples collected from Minnesota, Iowa and Illinois [32]. Furthermore, an indirect PDCoV S1 protein-based enzyme linked immunosorbent assay (ELISA) test suggested an even earlier presence of PDCoV IgG antibody in four archived serum samples collected in 2010 from US pigs [33]. In response to the large number of Swine Enteric Coronavirus Disease (SECD) cases since 2014, the United States Department of Agriculture (USDA) issued a federal order in June 2014, making SECD (caused by PEDV or PDCoV) reportable diseases, which was rescinded on 6 March 2018. According to the last SECD weekly situation report on 8 March 2018, 300 confirmed PDCoV positive premises and 88 presumptive positive premises cumulated between June 2014 and March 2018 and were distributed in 18 states within the US (https://www.aasv.org/pedv/SECD_Situation_Report_180308.pdf; accessed on: 15 October 2021). To date, PDCoV diarrhea occurs at a relatively low reported case rate according to the Swine Health Information Center (https://www.swinehealth.org/domestic-disease-surveillance-reports/; accessed on: 15 October 2021). 

In mainland China, PDCoV was first reported in 2015 [19,34,35,36]; however, retrospective studies have shown that PDCoV could have emerged as a swine pathogen as early as 2004 [19]. A recent PDCoV surveillance study reported that 94 (13.07%) of 719 porcine diarrhoea samples collected from 18 provinces in China from March 2016 to June 2018 were PDCoV-positive by RT-qPCR [37]. The complete S genes of 11 PDCoV strains were determined. Seven were expectedly grouped into the Chinese lineage. However, CH-WH-2017 (GenBank accession no. MK040451) exhibited a closer relationship to the US/Japan/South Korea lineage; CH-HA3-2017, CH-HA1-2017 and CH-HA2-2017 strains (GenBank accession no. MK040455, MK040453 and MK040454) were clustered into a new branch and separate from the Chinese lineage. The latter showed the closest relationship to the Vietnam/Laos/Thailand lineage [37]. As of October 2021, PDCoV has been detected in 26 provinces of China, including all swine producing areas [35,37,38,39,40,41,42]. From mid-March 2014 to January 2016, PDCoV related diarrhoea outbreaks were successively identified in Canada, South Korea, Thailand, Vietnam, and Laos [30,43,44,45]. Retrospective studies revealed that PDCoV has been circulating in Taiwan [46], Japan [47], Mexico [48] and Haiti [18] since 2011, late 2013, 2015 and 2014, respectively. PDCoVs or PDCoV-like δ-CoVs have not been detected in the Europe, South American, African, and Australian continents, although retrospective studies have been conducted in Brazil [49] and Spain [50] to identify CoVs in wild birds and pigs, respectively. 

### 3.2. Genetic Diversity of Global PDCoV

**Figure 3:**
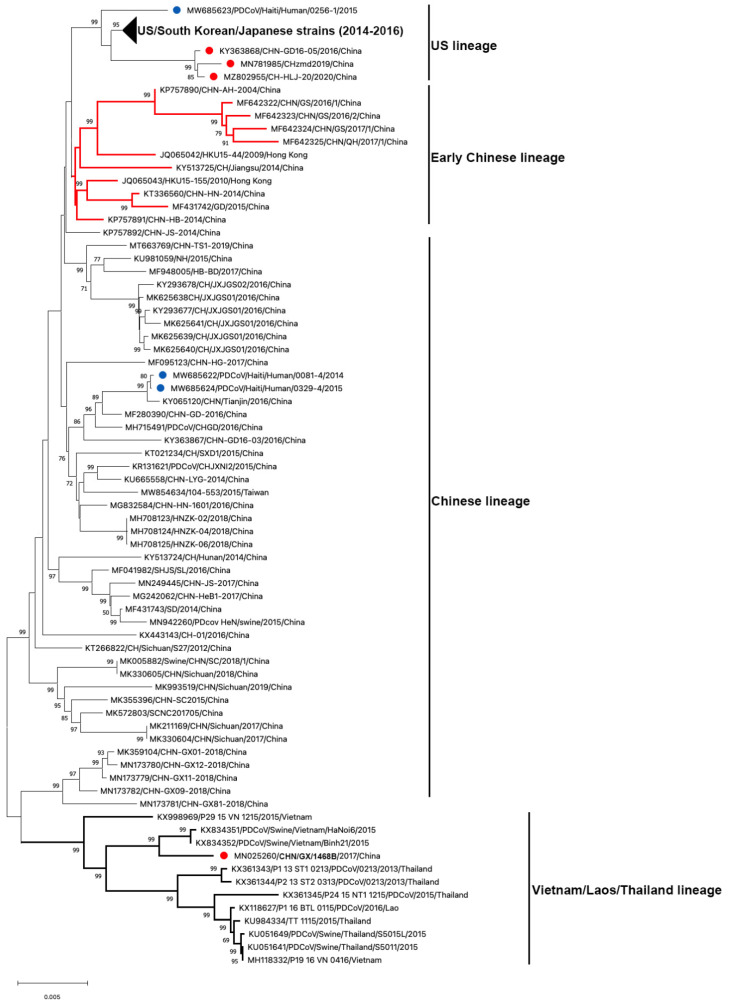
Phylogenetic analyses of PDCoV based on 122 complete genomes. Genome sequences were aligned with MAFFT v.7.490 [83]. The phylogenetic tree was constructed using the neighbour-joining method of MEGA 11, and bootstrap values (1000 replicates) above 70% are shown. The bar represents a corrected genetic distance. The red circles indicate PDCoV strains from China but that fall into non-Chinese lineages; the blue circles indicate PDCoV strains from humans in Haiti.

To date, 122 complete genome sequences of PDCoVs are available in GenBank (Table 1 and Figure 3). Phylogenetic analysis of these PDCoV genome sequences showed that all US, South Korean and Japanese strains clustered together; and three Chinese isolates (CHN-GD16-05/2016; CHzmd2019; CH-HLJ-20/2020) and one Haiti isolate (PDCoV/Haiti/Human/0256-1/2015) appeared to be more closely related to US/South Korean/Japanese strains. Therefore, these latter four strains were classified into the US linage (Figure 3). Similarly, He et al. found two other newly sequenced Chinese strains (AH2019/H and SD2019/426, GenBank accession no. are not available) were also clustered into the US lineage [10]. The close genetic relatedness between Chinese and US PDCoVs is not surprising due to the frequent trade of pork and pork products and pig feed supplements (https://www.fas.usda.gov/china-2020-export-highlights; accessed on: 10 November 2021). Additionally, it is not surprising to see that one of the three Haitian PDCoVs detected from children grouped within US lineage because pigs were re-introduced from the US to Haiti after the local pig population was mostly wiped out following the African Swine Fever epidemic in 1970s–1980s [51]. The early Chinese lineage contains the earliest strain that was detected from a sample collected in the Anhui Province in 2004 (CHN-AH-2004) and the strains isolated in Hong Kong in 2009 (HKU15-44) and 2010 (HKU15-155). The Chinese lineage is also inclusive of the two Haiti PDCoVs (PDCoV/Haiti/Human/0081-4/2014 and PDCoV/Haiti/Human/0329-4/2015) and the currently circulating strains in mainland China. The two Haitian strains are closely related to a Chinese strain (CHN/Tianjin/2016). According to the pork trade data from the Observatory of Economic Complexity (OEC), Chinese pork was exported to Haiti from 2006 to 2011 (https://oec.world/en/visualize/tree_map/hs92/import/hti/show/10203/2012/; accessed on: 10 November 2021). This may have contributed to PDCoV transmission from China to Haiti. The Southeast Asian (or Vietnam/Laos/Thailand) lineage contains PDCoV strains prevailing in these countries and a novel strain detected in China (CHN/GX/1468B/2017), indicating that these strains may derive from common evolutionary ancestors [52]. In addition, another Thailand-like PDCoV isolate (CHN-GX81-2018) was also discovered in Guangxi Province of China [53]. Collectively, phylogenetic analysis demonstrated that multiple PDCoV lineages, including US lineage, early Chinese lineage, Chinese lineage, and Vietnam/Laos/Thailand lineage, coexist in mainland China. He et al. [10] reported that more frequent intra- and inter-lineage recombination and higher virus genetic diversity were identified among and within the Vietnam/Laos/Thailand and Chinese lineages than within the US lineage. Frequent recombination events between different lineages of PDCoV strains in China may complicate PDCoV epidemiology and result in the emergence of PDCoV strains with changed pathogenicity and host tropism.

### 3.3. Co-Infection of PDCoV with Other Porcine Enteric Viruses 

Clinical signs associated with PDCoV infection were found to be less severe than PEDV infection, and the PDCoV prevalence rate in diarrheic pigs is about 30% [7,9,30]. Nevertheless, co-infection with other enteric viral pathogens, such as PEDV, transmissible gastroenteritis virus (TGEV) and porcine rotavirus (PRV), are common and may lead to more severe clinical disease [30,37,41,84,85]. Recent surveys conducted in the United States, Mexico, South Korea and mainland China found that PEDV is the most frequent co-infecting pathogen with PDCoV, with a detection rate ranging from 4.73% to 54.10% (Table 2). Furthermore, except for the US, multiple-infection cases with both PDCoV and two other viruses (PEDV, TGEV, or PRV) were identified in other countries, with a detection rate ranging from 0.12% to 18.80% (Table 2). Additionally, a recent study found the co-infection of PDCoV with 4 to 7 other viral pathogens in diarrhoea samples collected in China from 2015 to 2018. These pathogens were porcine astrovirus (PAstV), porcine teschovirus (PTV), porcine sapelovirus (PSV), porcine enterovirus (PEV) 9/10, torque teno sus virus 2 (TTSuV-2), mammalian reovirus (MRV), porcine torovirus (PToV), porcine kobuvirus (PKV) or porcine bocavirus (PBoV) (Table 2).

## 4. Cross-Species Transmission and Zoonotic Potential

### 4.1. Receptor Usage

To initiate infection, the interaction between CoV spike proteins and the host receptor(s) plays an important role and determines the extent of host cell susceptibility and interspecies transmission. Coronavirus spike proteins can bind to a wide variety of carbohydrates and protein receptors located at the cell surface, with receptor binding domains (RBDs) located in either the N-terminal domain (NTD) or the C-terminal domain (CTD) within the S1 subunit of the spike proteins [88]. Porcine aminopeptidase N (pAPN) has been identified as a PDCoV primary entry receptor [89,90]. PDCoV was shown to infect pig, human and chicken cells via APN and can use feline and human APN to infect nonpermissive cells [90]. The CTD of the S1 subunit of PDCoV spike protein binds to the interspecies conserved domain II of APN [90]. However, PDCoV may use additional receptors for cellular entry because pAPN knockout porcine intestinal epithelial (IPI-2I) cells can still be infected by PDCoV [91]. Furthermore, CD163 and pAPN double-gene-knockout pigs exhibited decreased susceptibility to PDCoV [92]. A recent study reported that sialic acids serve as a binding receptor of PDCoV [93]; however, whether sialic acids are PDCoV receptors or just attachment factors is still unknown. Additionally, lung fibroblast-like cells, derived from the pAPN knockout porcine alveolar macrophage (PAM) cultures, still supported PDCoV replication to high levels [94]. Similarly, Niu et al. [28] found that icPDCoV-RBD_ISU_ replicated to higher levels in APN knockout swine testis (ST) cells than in wild type ST cells, suggesting that the icPDCoV-RBD_ISU_ virus uses other unidentified host cell molecules, but not pAPN as the major receptor. In addition to receptor usage, other mechanisms might be involved in the cellular entry processes of PDCoVs. Recent studies have confirmed that macropinocytosis, clathrin-mediated endocytosis and pAPN-mediated endocytotic pathways play important roles in assisting PDCoV cellular entry and efficient viral replication [95,96,97].

### 4.2. Experimental Cross-Species Transmission of PDCoV 

The host tropism of coronaviruses is largely determined by the spike protein owing to its receptor binding and membrane fusion functions. A critical step in cross-species transmission of CoVs is the capacity to bind to new receptors in a novel host, which occurs by the spike protein [90]. Due to their large RNA genomes, ranging from 26 to 32 kilobases in length, CoVs are genetically more tolerant to acquire non-lethal mutations, insertions and deletions, and recombination in S gene, increasing CoV capacity to cross host species barriers. The S gene of the δ-CoV identified in Asian leopard cats and Chinese ferret badgers share over 99.80% nucleotide identity with PDCoV, suggesting a potential cross-species transmission between these wild mammals and pigs [7]. Additionally, a recent study reported that PDCoV was detected in plasma samples of three Haitian children with an acute undifferentiated febrile illness [18]. In addition to natural cross-species transmission, PDCoV experimentally infected 3–7-day-old gnotobiotic calves with a high level of viral RNA in faeces and PDCoV-specific IgG antibody in convalescent sera [17]. Recent studies showed that chickens and turkey poults were also susceptible to PDCoV infection and passed the virus efficiently to contact chicks [15,16]. Additionally, PDCoV successfully (productively) infected a number of cells from an unusually broad species range (Table 3). Interestingly, Niu et al. [28] found that the chimeric PDCoVs with SpDCoV spike protein (HKU17) or the RBD (ISU73347) infected pigs but lost its virulence and intestinal tropism but retained respiratory tropism suggesting limited potential for direct cross-species spill over of current sparrow δ-CoVs to pigs. These observations indicate that PDCoV has the potential to infect diverse host species. More surveillance and sequence data and seroprevalence studies are needed to understand the mechanisms of cross-species transmission of PDCoVs among different species.

### 4.3. PDCoV Zoonotic Potential

In the last two decades, three zoonotic CoVs (SARS-CoV, MERS-CoV, and SARS-CoV-2) have posed a serious threat to public health and global security [102,103]. In addition, HCoV-HKU1, HCoV-229E, HCoV- NL63, HCoV-OC43 and HECV-4408 are also of zoonotic origin [104,105,106,107,108]. Recently, Vlasova et al. [109] first characterized a complete genome of novel canine-feline recombinant alphacoronavirus (CCoV-HuPn-2018, GenBank accession no. MW591993.2) which was isolated from a child with pneumonia in Malaysia. This report was followed by another study that identified and characterized a highly similar canine-feline recombinant alphacoronavirus in a member of a medical team that travelled to Haiti [110]. In November 2021, Lednicky et al. [18] reported the first evidence that PDCoV may have jumped from pigs to three children in Haiti. The three children had mild symptoms (febrile, cough, and/or abdominal pain) and recovered. Genomic sequence analyses showed that Haiti/Human/0256-1/2015 and USA/Porcine/Arkansas61/2015 shared 99.40% nucleotide identity; and Haiti/Human/0081-4 and Haiti/Human/0329-4/2015 had 99.80% and 99.97% nucleotide identities with the CHN/ Porcine/Tianjin/2016, respectively [18]. These data suggest that at least two independent cross-species transmission events of PDCoV from pigs to humans occurred. It is still unknown whether PDCoV is transmissible among humans. Currently, PDCoV has only been demonstrated to cause asymptomatic or mild disease in humans and does not seem to pose a major health risk to human health. However, continued surveillance and seroprevalence studies of PDCoV in humans, especially those in close contact with pigs (e.g., swine farm families and workers and slaughterhouse workers), may provide a clearer answer in the future. Collectively, it appears inevitable that similar spill over events will occur again in the future, so more epidemiological studies are needed to clarify the origin, epidemiology, and cross-species transmission mechanisms of CoVs for improved CoV disease control and prevention. 

## 5. Conclusions, Challenges, and Future Perspectives

Coronaviruses are an important group of pathogens that can have a devastating impact on human and animal health, with new zoonotic CoVs continually emerging or re-emerging. PDCoV is an emerging pathogen of pigs that can also infect birds, and several mammalian species, including humans. PDCoV likely evolved directly or indirectly from avian δ-CoVs, although the exact origin pathway of PDCoV is still unknown. While its prevalence and disease severity are lower than those of PEDV, PDCoV co-infections with other porcine enteric pathogens are common, posing a threat to the swine industry. All PDCoV strains share >96.70% nucleotide identity at the genomic level, suggesting their recent emergence. Since the evidence of δ-CoVs infections of small carnivore mammals and humans is limited to one report, respectively, and these δ-CoVs are genetically indistinguishable from PDCoVs from pigs, it may indicate that PDCoVs are still in the process of adaption to different species, including humans. Currently, PDCoV strains form four lineages and all lineages of PDCoVs have been detected in China. The large pig population and low biosecurity in the swine farms in China [111,112] and the relatively mild diseases caused by PDCoVs can contribute to the frequent recombination and generation of new PDCoV variants, consequently facilitating interspecies transmission to other species. 

Although pAPN has been shown to be the major receptor for PDCoV infection, additional minor receptors exist that remain to be identified. There are still intriguing questions to be clarified about PDCoV, such as whether PDCoV could/can naturally infect other mammal species; whether frequent spill overs of PDCoV occur between birds and pigs and other mammals, such as MERS-CoV between camels and humans [113,114]; and whether PDCoV has spread to Europe, South America, Africa or Australia. These questions cannot be fully answered due to the unavailability of comprehensive epidemiological data. Therefore, additional studies to evaluate the genetic diversity of δ-CoVs in avian and mammalian hosts are needed to better understand their ecology and evolution. Furthermore, experimental studies on PDCoV interspecies transmission and host adaptation are needed to evaluate its pathogenicity in various hosts. Additionally, seroprevalence studies in pigs can provide useful information highlighting the actual prevalence of PDCoV globally. Finally, continued surveillance and seroprevalence studies of PDCoV in humans are needed to define its impact on public health. Safe and effective vaccines may be needed for pigs to prevent infection, to reduce the PDCoV gene pool that contribute to spill overs [18], and may be needed for humans if severe cases emerge.

## Figures and Tables

**Table 1:** Complete genome information on PDCoV strains.

Lineage	GenBank Accession No.	Strain Name	Countries	Collection Date	Host	Reference
US	KJ462462.1	OH1987	USA	31-Jan-2014	Pig	[9]
	KJ481931.1	PDCoV/USA/Illinois121/2014	USA	04-Jan-2014	Pig	[54]
	KJ567050.1	8734/USA-IA/2014	USA	20-Feb-2014	Pig	[55]
	KJ569769.1	IN2847	USA	13-Feb-2014	Pig	[56]
	KJ584355.1	IL2768	USA	12-Feb-2014	Pig	[57]
	KJ584356.1	SD3423	USA	20-Feb-2014	Pig	[57]
	KJ584357.1	KY4813	USA	07-Mar-2014	Pig	[57]
	KJ584358.1	PA3148	USA	18-Feb-2014	Pig	[57]
	KJ584359.1	NE3579	USA	21-Feb-2014	Pig	[57]
	KJ620016.1	MI6148	USA	18-Mar-2014	Pig	[57]
	KM012168.1	Michigan/8977/2014	USA	17-Mar-2014	Pig	Unpublished
	KR150443.1	USA/Arkansas61/2015	USA	24-Mar-2015	Pig	Unpublished
	KR265847.1	USA/Minnesota442/2014	USA	06-Mar-2014	Pig	Unpublished
	KR265848.1	USA/Minnesota214/2014	USA	14-Mar-2014	Pig	Unpublished
	KR265849.1	USA/Michigan447/2014	USA	02-Apr-2014	Pig	Unpublished
	KR265850.1	USA/Michigan448/2014	USA	02-Apr-2014	Pig	Unpublished
	KR265851.1	USA/Indiana453/2014	USA	13-May-2014	Pig	Unpublished
	KR265852.1	USA/Illinois449/2014	USA	21-Apr-2014	Pig	Unpublished
	KR265853.1	USA/Minnesota/2013	USA	14-Oct-2013	Pig	Unpublished
	KR265854.1	USA/Minnesota454/2014	USA	21-May-2014	Pig	Unpublished
	KR265855.1	USA/Minnesota455/2014	USA	21-May-2014	Pig	Unpublished
	KR265856.1	USA/Illinois272/2014	USA	23-Feb-2014	Pig	Unpublished
	KR265857.1	USA/Illinois273/2014	USA	23-Feb-2014	Pig	Unpublished
	KR265858.1	USA/NorthCarolina452/2014	USA	06-May-2014	Pig	Unpublished
	KR265859.1	USA/Minnesota159/2014	USA	11-Feb-2014	Pig	Unpublished
	KR265860.1	USA/Nebraska209/2014	USA	05-Feb-2014	Pig	Unpublished
	KR265861.1	USA/Nebraska210/2014	USA	05-Feb-2014	Pig	Unpublished
	KR265862.1	USA/Ohio444/2014	USA	26-Mar-2014	Pig	Unpublished
	KR265863.1	USA/Ohio445/2014	USA	27-Mar-2014	Pig	Unpublished
	KR265864.1	USA/Minnesota292/2014	USA	14-Mar-2014	Pig	Unpublished
	KR265865.1	USA/Iowa459/2014	USA	05-Jun-2014	Pig	Unpublished
	KT381613.1	OH11846	USA	07-May-2014	Pig	[58]
	KX022602.1	PDCoV/USA/Iowa136/2015	USA	15-Oct-2015	Pig	Unpublished
	KX022603.1	PDCoV/USA/Minnesota140/2015	USA	18-Dec-2015	Pig	Unpublished
	KX022604.1	PDCoV/USA/Nebraska137/2015	USA	27-Nov-2015	Pig	Unpublished
	KX022605.1	PDCoV/USA/Nebraska145/2015	USA	21-Dec-2015	Pig	Unpublished
	MZ291567.1	OH-FD22 P7	USA	2014	Pig	[59]
	KY354363.1	DH1	South Korea	01-Apr-2016	Pig	[60]
	KY354364.1	DH2	South Korea	01-Apr-2016	Pig	[60]
	KY364365.1	KNU16-07	South Korea	Jul-2014	Pig	[61]
	KY926512.1	KNU16-11	South Korea	Nov-2016	Pig	[62]
	KM820765.1	KNU14-04	South Korea	Apr-2014	Pig	[43]
	LC260038.1	AKT/JPN/2014	Japan	May-2014	Pig	[47]
	LC260039.1	GNM-1/JPN/2014	Japan	May-2014	Pig	[47]
	LC260040.1	GNM-2/JPN/2014	Japan	May-2014	Pig	[47]
	LC260041.1	IWT/JPN/2014	Japan	May-2014	Pig	[47]
	LC260042.1	MYZ/JPN/2014	Japan	May-2014	Pig	[47]
	LC260043.1	OKN/JPN/2014	Japan	Aug-2014	Pig	[47]
	LC260044.1	YMG/JPN/2014	Japan	Dec-2014	Pig	[47]
	LC260045.1	HKD/JPN/2016	Japan	Sep-2016	Pig	[63]
	KY363868.1	CHN-GD16-05	China	05-Jan-2016	Pig	[64]
	MN781985.1	CHzmd2019	China	Unknown	Pig	Unpublished
	MZ802955.1	CH-HLJ-20	China	Sep-2020	Pig	Unpublished
	MW685623.1	PDCoV/Haiti/Human/0256-1/2015	Haiti	16-Mar-2015	Human	[18]
Early Chinese	KP757890.1	CHN-AH-2004	China	24-May-2004	Pig	[19]
	KP757891.1	CHN-HB-2014	China	26-Dec-2014	Pig	[19]
	JQ065042.2	HKU15-44	China: Hong Kong	2009	Pig	[7]
	JQ065043.2	HKU15-155	China: Hong Kong	2010	Pig	[7]
	KT336560.1	CHN-HN-2014	China	24-Nov-2014	Pig	[65]
	KY513725.1	CH/Jiangsu/2014	China	2014	Pig	Unpublished
	MF431742.1	GD	China	2015	Pig	Unpublished
	MF642322.1	CHN/GS/2016/1	China	Aug-2016	Pig	[66]
	MF642323.1	CHN/GS/2016/2	China	Aug-2016	Pig	[66]
	MF642324.1	CHN/GS/2017/1	China	Apr-2017	Pig	[66]
	MF642325.1	CHN/QH/2017/1	China	Mar-2017	Pig	[66]
Chinese	KP757892.1	CHN-JS-2014	China	20-Dec-2014	Pig	[19]
	KR131621.1	PDCoV/CHJXNI2/2015	China	Mar-2015	Pig	[35]
	KT021234.1	CH/SXD1/2015	China	20-Mar-2015	Pig	[34]
	KT266822.1	CH/Sichuan/S27/2012	China	2012	Pig	[36]
	KU665558.1	CHN-LYG-2014	China	26-Jun-2014	Pig	Unpublished
	KU981059.1	NH	China	16-Feb-2015	Pig	Unpublished
	KX443143.2	CH-01	China	2016	Pig	[67]
	KY065120.1	CHN/Tianjin/2016	China	2016	Pig	[68]
	KY293677.1	CH/JXJGS01/2016	China	23-May-2016	Pig	[69]
	KY293678.1	CH/JXJGS02/2016	China	23-May-2016	Pig	Unpublished
	KY363867.1	CHN-GD16-03	China	18-Mar-2016	Pig	[64]
	KY513724.1	CH/Hunan/2014	China	2014	Pig	Unpublished
	MF041982.1	SHJS/SL/2016	China	23-Dec-2016	Pig	[70]
	MF095123.1	CHN-HG-2017	China	15-Feb-2017	Pig	[71]
	MF280390.1	CHN-GD-2016	China	2016	Pig	[72]
	MF431743.1	SD	China	2014	Pig	Unpublished
	MF948005.1	HB-BD	China	10-Aug-2017	Pig	[73]
	MG242062.1	CHN-HeB1-2017	China	2017	Pig	Unpublished
	MG832584.1	CHN-HN-1601	China	Jul-2016	Pig	[74]
	MH708123.1	HNZK-02	China	20-Mar-2018	Pig	[67]
	MH708124.1	HNZK-04	China	20-Mar-2018	Pig	[75]
	MH708125.1	HNZK-06	China	20-Mar-2018	Pig	[75]
	MH715491.1	PDCoV/CHGD/2016	China	2016	Pig	Unpublished
	MK005882.1	Swine/CHN/SC/2018/1	China	Mar-2018	Pig	Unpublished
	MK211169.1	CHN/Sichuan/2017	China	24-Dec-2017	Pig	Unpublished
	MK330604.1	CHN/Sichuan/2017	China	Feb-2017	Pig	Unpublished
	MK330605.1	CHN/Sichuan/2018	China	Jan-2018	Pig	Unpublished
	MK355396.1	CHN-SC2015	China	20-Feb-2016	Pig	[76]
	MK359104.1	CHN-GX01-2018	China	2018	Pig	[53]
	MK572803.1	SCNC201705	China	Jun-2017	Pig	Unpublished
	MK625638.1	CH/JXJGS01/2016	China	Oct-2018	Pig	[69]
	MK625639.1	CH/JXJGS01/2016	China	Oct-2018	Pig	[69]
	MK625640.1	CH/JXJGS01/2016	China	Oct-2018	Pig	[69]
	MK625641.1	CH/JXJGS01/2016	China	Oct-2018	Pig	[69]
	MK993519.1	CHN/Sichuan/2019	China	Jan-2019	Pig	Unpublished
	MN173779.1	CHN-GX11-2018	China	2018	Pig	[53]
	MN173780.1	CHN-GX12-2018	China	2018	Pig	[53]
	MN173781.1	CHN-GX81-2018	China	2018	Pig	[53]
	MN173782.1	CHN-GX09-2018	China	2018	Pig	[53]
	MN249445.1	CHN-JS-2017	China	11-Dec-2017	Pig	[77]
	MN942260.1	PDCoV HeN/swine/2015	China	2015	Pig	Unpublished
	MW854634.1	104-553	China: Taiwan	Jun-2015	Pig	[46]
	MT663769.1	CHN-TS1-2019	China	23-Jul-2019	Pig	Unpublished
	MW685622.1	PDCoV/Haiti/Human/0081-4/2014	Haiti	15-Dec-2014	Human	[18]
	MW685624.1	PDCoV/Haiti/Human/0329-4/2015	Haiti	13-Apr-2015	Human	[18]
Vietnam/ Laos/ Thailand	KU051641.1	PDCoV/Swine/Thailand/S5011/2015	Thailand	10-Jun-2015	Pig	[78]
	KU051649.1	PDCoV/Swine/Thailand/S5015L/2015	Thailand	30-Jun-2015	Pig	[78]
	KU984334.1	TT_1115	Thailand	Nov-2015	Pig	[79]
	KX118627.1	P1_16_BTL_0115/PDCoV/2016/Lao	Laos	20-Jan-2016	Pig	[44]
	KX361343.1	P1_13_ST1_0213/PDCoV/0213/Thailand	Thailand	Feb-2013	Pig	[80]
	KX361344.1	P2_13_ST2_0313/PDCoV/0213/Thailand	Thailand	Mar-2013	Pig	[80]
	KX361345.1	P24_15_NT1_1215/PDCoV/2015/Thailand	Thailand	Dec-2015	Pig	[80]
	KX834351.1	PDCoV/Swine/Vietnam/HaNoi6/2015	Vietnam	10-Oct-2015	Pig	[81]
	KX834352.1	PDCoV/Swine/Vietnam/Binh21/2015	Vietnam	08-Dec-2015	Pig	[81]
	KX998969.1	P29_15_VN_1215	Vietnam	Dec-2015	Pig	Unpublished
	MH118332.1	P19_16_VN_0416	Vietnam	Unknown	Pig	[82]
	MN025260.1	CH/GX/1468B/2017	China	18-Jan-2017	Pig	[52]

**Table 2:** Co-infection of PDCoV with other porcine enteric viruses in pig farms or diarrheic samples from 2012 to 2018.

Year	Country	Pathogens	Positive Farms/Samples (Positive Rate)	References
2014–2018	United States	PDCoV + PEDV	307	[86]
2014–2017	Mexico	PDCoV + PEDV	46 (54.1%)	[48]
		PDCoV + TGEV	9 (10.6%)	
		PDCoV + PEDV + TGEV	16 (18.8%)	
2014–2016	South Korea	PDCoV + PEDV	43 (6.3%)	[62]
		PDCoV + PRV	19 (2.78%)	
		PDCoV + PEDV + PRV	2 (0.29%)	
2016–2018	China	PDCoV + PEDV	34 (4.73%)	[37]
2012–2018		PDCoV + PEDV	380 (46.74%)	[41]
		PDCoV + TGEV	30 (3.69%)	
		PDCoV + PEDV + PRV	3 (0.37%)	
		PDCoV + PEDV + TGEV	1 (0.12%)	
2015–2018		PDCoV + PAstV + PEV + TTSuV-2 + MRV + PKV	1 (1.12%)	[87]
		PDCoV + PAstV + PEV + PKV + PBoV	1 (1.12%)	
		PDCoV + PAstV + PTV + PSV + PEV + PKV + PBoV	1 (1.12%)	
		PDCoV + PAstV + PTV + PSV + PKV + PBoV	1 (1.12%)	
		PDCoV + PAstV + PTV + PSV + PEV + TTSuV-2 + PKV + PBoV	1 (1.12%)	
		PDCoV + PAstV + PTV + PEV + TTSuV-2 + PToV + PBoV	1 (1.12%)	
		PDCoV + PAstV + PEV + TTSuV-2 + PBoV	1 (1.12%)	

**Table 3:** Cells susceptible to PDCoV infection in vitro.

Cell or Cell Line	Origin	References
LLC-PK1	Porcine kidney proximal tubular epithelials cells	[59]
ST	Swine testicular cells	[59]
IPEC-J2	Porcine small intestinal epithelial cells	[98]
IPI-2I	Porcine ileum epithelial cells	[99]
IPAM	Immortalized porcine cell line of pulmonary alveolar macrophages	[100]
Huh7	Human hepatoma cells	[90]
A549	Human lung carcinoma cells	[100]
LMH	Chicken leghorn male hepatoma cells	[90]
DF-1	Chicken fibroblast cells	[90]
White-feather broiler embryos	White-feather broiler	[16]
Hyline layer embryos	Hyline layer	[16]
PBK	Primary bovine kidney cells	[101]
PBH	Primary bovine heart cells	[101]
MDBK	Madin Darby bovine kidney cells	[100]
Vero	African green monkey kidney epithelial cells	[100]

## Data Availability

The data presented in this study are openly available at GenBank (https://www.ncbi.nlm.nih.gov/nucleotide/, accessed on: 15 October 2021) and the websites provided in this manuscript.
